# The Role of Attention in Dyadic Social Interaction With Infants and Their Caregivers

**DOI:** 10.1111/infa.70110

**Published:** 2026-07-16

**Authors:** Olivia Boorom, Brenda Salley

**Affiliations:** ^1^ Department of Speech‐Language‐Hearing: Sciences and Disorders University of Kansas Lawrence Kansas USA; ^2^ Department of Communication Sciences and Disorders Georgia State University Atlanta Georgia USA; ^3^ Department of Pediatrics University of Kansas Medical Center Kansas City Kansas USA

**Keywords:** attentional skills, interaction dynamics, parent–child interaction, synchrony

## Abstract

This study examined the relationship among early attention skills in infants and measures of dyadic interaction during caregiver–infant play, including interpersonal synchrony and turn‐taking behavior. Thirty‐five caregivers and their one‐year‐old infants (mean age = 15.34 months) completed a 5‐minute free play session and sessions were coded for communication behaviors (eye contact, vocalizations, and gestures), coordinated attention, and infant visual attention states (focused, unfocused, and casual attention). Coordinated attention with a caregiver was not related to caregiver–infant synchrony and turn‐taking. Mean duration of casual attention (i.e., passive, exploratory attention toward an object or person), but not focused attention, was negatively associated with time spent in bouts of turn‐taking. Results suggest that shorter bouts of casual attention during play may support longer periods of turn‐taking with caregivers, an important social communication skill for infants as intentional communication continues to develop in the second year of life.

## 1 Introduction

In the first year of life, developing visual attention skills allow infants to access and gain information from the world around them, including during social interactions with others. There is a significant body of research linking attention skills in infancy to later language and communication development (Deák et al. 2013; Kannass and Oakes 2008; Lawson and Ruff 2004; Salley et al. 2013, 2016; Wagner et al. 2013); however, the mechanisms through which attention skills support language development are still unclear. Observable patterns of attention during naturalistic interactions may provide a window into how infants' attention supports language and communication in the social context. The goal of the current study was to examine how infant attention during naturalistic caregiver–child play is associated with measures of social interaction, which may have downstream effects on later language development.

### 1.1 Visual Attention Development

Infant visual attention development lays an important foundation for social communication skills and language development. Infants develop spatial orienting skills and attention to object features such as color and shape in the first 6 months of life (Colombo 2001). From 3 to 12 months, infants also develop increased ability to modulate their attention, by inhibiting, shifting, or sustaining their attention to a stimulus (Colombo 2001). The ability to modulate attention, that is, endogenous attentional skills, supports the development of coordinated attention with communication partners and objects toward the end of the first year of life (Bakeman and Adamson 1984; Courage et al. 2006; Deák et al. 2013; Ruff and Capozzoli 2003). Importantly, sustained attention and coordinated attention are maturing at the same time as infants are developing pivotal social communication skills such as gestures, babbling, and intentional communication (Deák et al. 2013; Iverson and Goldin‐Meadow 2005).

There is a well‐established body of research investigating how these attentional processes support language development by allowing infants to follow their caregiver's attention and map words labels to objects (Bakeman and Adamson 1984; Bottema‐Beutel 2016; Kasari et al. 2008; Salley et al. 2013; Tomasello and Farrar 1986). Here we focus specifically on coordinated attention skills, wherein two communication partners are attending to the same object or event at the same time. We use the term coordinated attention to be inclusive of several types of joint attention configurations; for example, caregivers and infants may both attend to the same toy without alternating their gaze toward each other to “check in,” or infants may use triadic gaze to direct their caregiver's attention toward a toy that they want. An expanded discussion of differences in the conceptualization and operationalization of joint attention in the literature can be found in Abney et al. (2020). Importantly, coordinated attention in infants as young as 9 months is positively related to later language outcomes (Abney et al. 2020; Tamis‐LeMonda et al. 2001; Tomasello and Farrar 1986), and may serve as a bridge between infants developing visual attention skills and language learning.

### 1.2 Coordinated and Sustained and Attention Support Language Development

Recent work has investigated how visual attention supports dyadic reciprocal interactions, which may also have downstream effects on language development (Abney et al. 2020; Hoehl and Bertenthal 2021; Schroer and Yu 2022). This growing body of literature considers the bidirectional processes that drive real‐time changes in attention and opportunities for language learning. For instance, during caregiver–infant interactions caregivers play an important role in maintaining coordinated attention episodes by using triadic gaze to attend to both the object and the infant and naming the object that the infant is attending to (Abney et al. 2020). There is also evidence that relationships between infant and caregiver attention are bidirectional, rather than driven solely by the caregiver. Sustained infant visual attention during joint play is associated with greater activity in neural frequency bands associated with attention in caregivers, suggesting that infant attention may drive real‐time changes in caregiver neural responsiveness and attention (Wass et al. 2018).

The sustained attention hypothesis suggests that the underlying mechanism through which coordinated attention supports vocabulary development is through sustained attention to objects (Yu et al. 2019). Specifically, this theory hypothesizes that episodes of coordinated attention work to extend infants visual attention to objects, allowing for more successful object‐label mapping. In fact, there is evidence suggesting that time spent looking at objects and events in the presence of distractors at 11 months is directly associated with later vocabulary at 18 months, even after controlling for initial language skills (Salley et al. 2013). As early as 7 months old, focused attention to objects predicts executive function and early cognitive skills later in childhood in infants born prematurely (Lawson and Ruff 2004). Additionally, time spent in sustained attention during episodes of coordinated attention, rather than the coordinated attention episodes themselves, more strongly predicts later vocabulary skills in the second year of life (Yu et al. 2019). Infants indeed spend most of their time in naturalistic play attending to objects rather than communication partners (Abney et al. 2020; Mason et al. 2019; Suarez‐Rivera et al. 2019), though there is evidence that social context and coordinated attention with a communication partner may scaffold infants' attention to support language learning (Abney et al. 2020; Schroer and Yu 2022). Caregiver communication acts such as labeling, touch, and triadic joint attention lead to longer bouts of infant sustained attention (Suarez‐Rivera et al. 2019), but importantly the presence of coordinated visual attention with a caregiver is associated with longer durations of sustained attention even without the addition of other communicative behaviors (Yu and Smith 2016).

Schroer and Yu (2022) expanded on this work by proposing that infant attention moderates the relationship between parent responsiveness and cognitive and language outcomes. They found that infant attention episodes were longer when accompanied by parent speech and the effect of parent speech on attention duration was stronger in parents who were less talkative (Schroer and Yu 2022). One possible explanation for the differences between less talkative and more talkative parents is that parents who are less talkative may be more selective in their speech, choosing opportunities to label, comment on, or maintain their child's attention that are more salient (Schroer and Yu 2022). While more research is needed to disentangle individual differences in parent responsiveness, this work demonstrates one pathway by which responsiveness supports infant language learning in real time, by extending infant visual attention.

### 1.3 Attention as a Potential Facilitator of Dyadic Interaction

Beyond labeling to support object‐label mapping, extending infant visual attention may allow for increased opportunities for social interaction. Gartstein et al. (2008) investigated this relationship by assessing whether two dyadic interaction factors—responsivity/sensitivity and synchrony/reciprocity—predicted infant duration of visual orienting. Dyadic responsivity/sensitivity and infant vocal reactivity significantly predicted infant perceptual sensitivity, a measure of attention to low intensity stimuli in the environment (Gartstein et al. 2008). Additionally, synchrony/reciprocity was significantly associated with shorter durations of visual attention. The authors hypothesized that parent–infant synchrony may be more closely linked to more flexible attentional states than focused attention, such that the ability to shift attention in social situations with a partner is more closely related to parent–infant reciprocity and turn‐taking (Gartstein et al. 2008). One way to measure more flexible attentional states is by examining casual attention. Casual attention is a state of passive attention to objects and people that contrasts with focused attention (Lansink et al. 2000; Ruff and Lawson 1990). Infants in states of casual attention may be engaged with an activity but in an inattentive manner, and casual attention often occurs at the beginning or ending of toy play, signaling a shift in engagement (Lansink et al. 2000). However, there is limited evidence for the relationship between different forms of attention such as focused versus casual attention and dyadic interaction variables. In the current study, we focus specifically on two measures of dyadic interaction that can be observed during naturalistic interactions: caregiver–infant interactional synchrony and turn‐taking. Interactional synchrony is a measure of congruence between two partner's behavior, or in other words, the degree to which communication partners align their behavior in time, over the course of an entire interaction (Davis et al. 2018; Provenzi et al. 2018). Synchrony can be measured using time‐series analytic techniques that account for timing and congruence, including correlation indices, shared variance, and cross‐recurrence quantification analysis (CRQA), among others (Leclère et al. 2014; Provenzi et al. 2018; Xu et al. 2020). Turn‐taking describes the organization of conversation, or how communication partners exchange their verbal and nonverbal actions (i.e., turns) between each other to create back‐and‐forth conversation (Cosper and Pika 2025; Schegloff 2007). Turn‐taking can be measured by the amount of time caregivers and infants spend in “bouts” of continuous back‐and‐forth interaction (Cosper and Pika 2025; Zhang et al. 2024). While CRQA can summarize the degree of synchrony over an entire play interaction, measuring turn‐taking allows us to look at individual “synchronous” behaviors between caregivers and their infants. Higher rates of turn‐taking during the first and second year of life is associated with improved language and developmental outcomes as much as 10 years later (Donnelly and Kidd 2021; Gilkerson et al. 2018; Zhang et al. 2024). Individual bouts of turn‐taking also present opportunities for language learning, and caregivers can promote and extend turn‐taking with their infant by labeling, commenting, and directing their behavior, much in the same way that caregivers extend infants' visual attention (Schroer and Yu 2022; Suarez‐Rivera et al. 2019; Zhang et al. 2024). These measures complement and expand on the subjective rating scale used in Gartstein et al. (2008) by quantifying the temporal and the behavioral aspects of synchrony and reciprocity using observational behavior coding.

Periods of social interaction are an important context for language learning, which can be scaffolded by caregivers through responsive communication and coordinated attention (Fogel and Thelen 1987; Thelen and Bates 2003). Synchrony during social interaction is also an important predictor of later communication and language outcomes for infants in the first year of life (Feldman and Greenbaum 1997; Harrist and Waugh 2002; Kellerman et al. 2020). Previous literature points to cascading effects among attentional, language, and social interaction skills in infancy (Oakes 2023). As infants shift from preintentional to intentional communication, between 9 and 12 months of age, they begin to play a greater role in sustaining and directing social interactions (Xu et al. 2020). Infants begin to adapt to the timing and turn‐taking patterns of their caregiver (Abney et al. 2017; Hilbrink et al. 2015) and caregivers begin to respond to infant social bids, such as eye gaze, such that infant communicative acts predict parent behavior during play. During this period turn‐taking becomes an increasingly important context for speech and language development, as infants engage in more speech‐like babbling during turn‐taking than they do during solo vocal play (Long et al. 2022). Infants, in turn, begin to adopt adult‐like patterns of gaze shifting during social interaction by the end of the second year of life (Rutter and Durkin 1987). Social attention skills such as alternating gaze, gaze following, attention to social stimuli, and mutual gaze are related to task‐based measures of social communication or characteristics of conditions associated with social communication difficulties, such as autism (Bedford et al. 2012; Nyström et al. 2017; Thorup et al. 2018; Wagner et al. 2013). Additionally, while there has been work exploring the role of visual attention to social stimuli, such as face‐looking and mouth‐looking, in infant language and social communication development (see Çetinçelik et al. 2021 for review), less is known about how other attention skills support dyadic social communication in real time during naturalistic interaction. Given the relationship between sustained and coordinated attention during caregiver–infant interactions (Schroer and Yu 2022; Yu and Smith 2016) and evidence that sustained attention is associated with larger reciprocal processes such as caregiver–infant synchrony (Gartstein et al. 2008), the current study seeks to further disentangle the role of infant attention in caregiver–infant communication using an interactionist lens. Understanding the role that attention plays during social interaction may reveal the processes by which attention supports communication development and word‐learning.

### 1.4 The Current Study

The purpose of the current study is to investigate whether early infant attention skills are associated with caregiver–infant interactional synchrony and turn‐taking skills. Both synchrony and turn‐taking during naturalistic interactions are positive indicators of attachment, language development, and social communication development in infants and toddlers (Feldman 2007; Feldman and Greenbaum 1997; Hilbrink et al. 2015). There is ample evidence suggesting that early attentional skills, particularly duration of orienting and focused attention, are related to later language outcomes (Kannass and Oakes 2008; Yu et al. 2019; Yu and Smith 2016). The current study aims to extend this work by investigating dyadic interaction skills as a potential intermediary skill linking early attention and language development in infancy. By investigating attention and dyadic interaction skills in 1‐year old infants and their caregivers, we were able to zero in on a period of development where joint attention and intentional communication skills are developing at the onset of spoken language. Because social engagement and reciprocal interaction provide an important context for language learning, periods of turn‐taking and synchrony may be a mechanism by which attention can facilitate language learning, leading to the improved language and vocabulary outcomes seen in previous work (Kannass and Oakes 2008; Yu et al. 2019).

First, this study aimed to assess how duration of coordinated attention is associated with caregiver–infant synchrony and turn‐taking. Because coordinated attention has been shown to extend infant attention to objects and play (Abney et al. 2020; Suarez‐Rivera et al. 2019; Yu and Smith 2016), periods of coordinated attention may also extend opportunities for reciprocal interaction around a shared focus of attention. We hypothesized that duration of coordinated attention would be positively associated with caregiver–infant synchrony and turn‐taking. Second, we aimed to explore how focused, casual, and unfocused attention are associated with caregiver–infant synchrony and turn‐taking. Infant visual attention skills have been hypothesized to drive positive outcomes associated with joint attention skills, therefore visual attention may be directly associated with social interaction outcomes such as synchrony and turn‐taking. Between the first and second year of life, infants are rapidly developing both endogenous attention skills which may cascade to their simultaneously developing social communication and language skills, and prior research has demonstrated concurrent associations between attention and language in this age group (Salley et al. 2016; Wagner et al. 2013). If focused attention is associated with caregiver–infant synchrony and turn‐taking, these findings would provide further support for the sustained attention hypothesis and offer new evidence that social interaction skills may mediate the link between sustained visual attention and later language skills (Yu et al. 2019). On the other hand, if casual attention is associated with infant synchrony and turn‐taking, this would suggest that the casual attention state may be flexible enough to allow for opportunities for reciprocal communication and joint attention between an object and the caregiver compared to focused or unfocused attention (Gartstein et al. 2008).

## 2 Method

### 2.1 Participants

Participants included 35 mothers and their 1‐year‐old typically developing infants (mean age = 15.34 months, range = 10.98–23.2 months). Participants were recruited for a larger longitudinal study of attention and language development by mail, email, and phone through listservs, newsletters, and research registries at University of Kansas Medical Center and Children's Mercy Hospital in Kansas City. A subset of 35 mother–infant dyads participating in the larger study were included in the current study. Participants were excluded if they demonstrated concerns for speech, motor, or developmental delays at the time of the study visit or any subsequent visits. Infant language skills were measured via parent report during the research center visit using the Receptive‐Expressive Emergent Language Test‐Third Edition (REEL‐3; Bzoch et al. 2003). The REEL‐3 is a norm‐referenced caregiver interview of their child's receptive and expressive language skills. The study was conducted in accordance with the ethical standards of the American Psychological Association and approved by the Human Subjects Committee at the University of Kansas Medical Center (#2932). All caregivers provided informed consent at the time of enrollment. Demographic characteristics of mothers and infants are described in Table 1.

**TABLE 1:** Participant demographics.

	Infants	Mothers
	*n* = 35	*n* = 35
Sex		
Male (*n*)	14	0
Female (*n*)	21	35
Race		
Black (*n*)	2	2
White (*n*)	33	31
More than one race (*n*)	7	2
Ethnicity		
Hispanic/Latino (*n*)	3	2
Not Hispanic/Latino (*n*)	32	33
Maternal education		
High school (*n*)	—	3
Two‐year postsecondary/some college (*n*)	—	9
Four‐year postsecondary (*n*)	—	17
Graduate and/or professional degree (*n*)	—	6
REEL‐3 language ability score (*n* = 33)		
Mean (SD)	90.39 (11.26)	—
Range	66–121	—

*Note:* Language ability scores are standard scores with a mean of 100. Average language ability scores range from 90 to 110.

Abbreviation: REEL‐3, Receptive‐Expressive Emergent Language Test‐Third Edition.

### 2.2 Procedures

#### 2.2.1 Caregiver–Child Free Play Interaction

**FIGURE 1:**
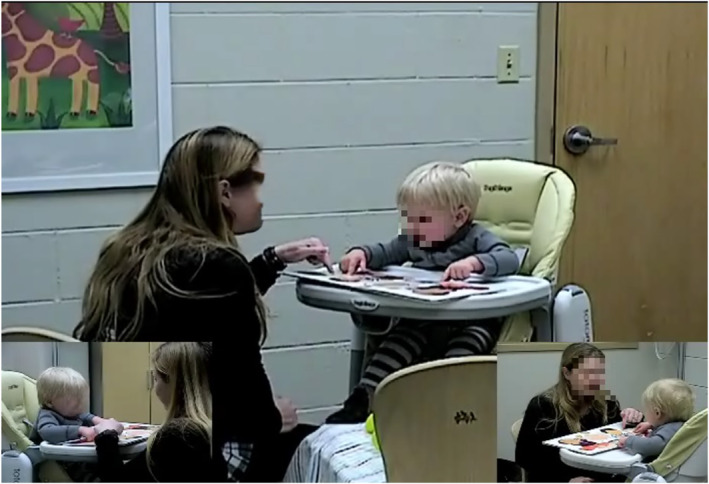
Caregiver–child free play configuration.

Mother–infant dyads participated in a caregiver–child free play interaction with a standard set of toys at the research center. Mothers and infants were instructed to play for 5 minutes. Infants were seated in a highchair while mothers were seated directly across from them with a set of toys. The face‐to‐face seated positioning was chosen because a primary aim of the caregiver–child free play interaction was to measure infant visual attention, and the seated positioning allowed for a more consistent, clear view of both the mother and infant's gaze direction. Toys provided included a baby doll, play phone, train, shape puzzle, stacking cups, a board book, and a puppet. Interactions were video and audio recorded for offline observation. Figure 1 shows a de‐identified example of the caregiver–child free play configuration.

#### 2.2.2 Observational Coding

Participant's caregiver–child free play interactions were coded offline using a continuous timed‐event coding scheme with Noldus Observer XT (Noldus 1991). Any portion of the session where either the mother or infant's upper body was out of view was considered uncodable. In addition, if there were any interruptions (e.g., phone ringing or examiner entering the session), the duration of the interruption was considered uncodable. Sessions were coded in two phases by separate coders. First, sessions were coded for infant attention states (unfocused, casual, or focused attention). Second, potentially communicative behavior from both parents (vocalizations and gestures) and infants (vocalizations, gestures, and eye contact) and periods of coordinated attention were coded. Coders marked the onset and offset of each event. All state‐based measures (i.e., infant attention states and coordinated attention) and interval‐based measures (infant and mother communication) are described as the proportion of time spent in each state out of the amount of codable time to account for differences in sample length.

##### 2.2.2.1 Infant Attention States

Caregiver–infant interactions were also coded for infant attention states using an observational coding scheme adapted from Ruff and Lawson (1990). All codable periods of the interaction were categorized as either (a) unfocused, undirected attention, (b) casual attention, or (c) focused attention, which were mutually exclusive and exhaustive. These attention states are frequently used to assess visual attention during naturalistic social tasks (Lansink et al. 2000; Lawson and Ruff 2004; Petrie Thomas et al. 2012). Brief attention shifts less than 1 second in duration were not considered changes in attention states. Rather, clear behavioral indicators of a shift between states were necessary to mark changes from one state to another (e.g., infant shifts from mouthing toy (casual) to looking around the room (unfocused) by taking their mouth off of the toy and turning their head). Unfocused attention occurred when the infant's focus and actions were not directed toward an object or their mother (e.g., infant is looking around the room or shifting quickly between many activities). Casual attention occurred when infants demonstrated visual attention toward an object or their mother, but they were engaging in exploratory play rather than goal‐directed play or clear play routines (e.g., mouthing a book). Focused attention occurred when the infant was visually attending to an object or their mother and engaging using purposeful and goal‐directed actions (e.g., using toys as intended, playing peek‐a‐boo with mother). The primary difference between focused and casual attention can be described as the difference between active engagement and passive engagement (Ruff and Lawson 1990). For analyses of infant attentional state, we calculated the total duration of time spent in each attentional state and the mean duration of periods of attention across each type of attentional state. The mean duration of each attention state thus reflects both the length of time spent in each period of attention, but may also reflect the degree to which infants switched attention or moved between attentional states. A subset of 28.6% of videos (10 of 35) were randomly selected by the primary coder and independently coded by a trained research assistant for reliability of infant attention state coding. Reliability coding was interspersed throughout the duration of the behavior coding procedures and percent agreement was monitored intermittently by the primary coder to check for coder drift. Percent agreement of attention state was 91.88% and the Kappa agreement value for classification of attention state was *κ* = 0.87.

##### 2.2.2.2 Coordinated Attention

Coordinated attention was coded in a second review of the mother–child interaction recordings by separate research staff from those that coded infant attention states. Coordinated attention was defined as a period of time in which both the mother and infant are attending to the same object or play, that is, demonstrating a shared focus of attention (Abney et al. 2020; Bakeman and Adamson 1984; Suarez‐Rivera et al. 2019; Tomasello and Farrar 1986). Coordinated attention could be indicated through talk (e.g., mother commenting on a toy the infant is playing with), touch (e.g., moving or using an object for at least 1 second), and/or gaze (e.g., clear unambiguous visual attention to an object for at least 1 second). Onsets and offsets for coordinated attention were determined by identifying clear behavioral indicators of changes in coordinated attention that lasted > 1 second, such as gaze shifts, touch, or talk about a common focus of attention. For analyses of coordinated attention, we examined the proportion of codable time spent in coordinated attention. Coordinated attention was represented as duration of coordinated attention states, and intra‐class correlation (ICC) values were calculated to assess interrater reliability. One‐way ICC values were 0.73 for coordinated attention, suggesting sufficient reliability among raters.

##### 2.2.2.3 Caregiver and Infant Communication

Caregiver's gestures and vocalizations were coded as communicative behavior. Eye gaze was not considered as a maternal communicative behavior because the face‐to‐face positioning of the mother–infant interaction resulted in high frequencies of sustained eye gaze toward the infant. Infants' gestures, vocalizations, eye gaze, and imitative play were all coded as potentially communicative behavior. Each of these behaviors were coded regardless of communicative intent or directedness because undirected communication may still be used as a jumping off point for a bout of turn‐taking with a responsive partner (Van Keer et al. 2019; Yirmiya et al. 2006). Onset and offset for all vocalizations were determined both perceptually by identifying the onset of speech and also by using the waveform visualization tool in Noldus Observer XT (Noldus 1991). Vocalizations included any non‐vegetative vocal behavior (examples of vegetative vocal behavior includes laughing, coughing, expressive sighs or raspberries) from either the caregiver or infant. These could include longer phrases, words, babbling, vowel sounds, or cooing. Segmentation of vocal behavior followed breath‐group procedures outlined by Nathani and Oller (2001). Gestures were defined as manual or body movements/configurations that carried communicative meaning to regulate behavior, gain attention, and/or represent words or concepts (Crais et al. 2004; Iverson and Goldin‐Meadow 2005). Coders were given a list of common conventional and representational gestures aligned with previous literature on infant and toddler gesture development (Crais et al. 2004). Frequently occurring gestures in this sample included giving, holding out an open hand to request, pointing, shrugging, shaking head no, and pantomiming an action. Onset of all gestures was determined by identifying when the hand or body configuration of the gesture became recognizable (e.g., when caregiver index finger is extended enough to perceive a discernible point) or at the first discernible motion of more dynamic gestures (e.g., child head moves fully to one side to begin a head shake). Offsets of gesture were identified similarly, at the moment when the hand or body configuration no longer took the recognizable shape of the gesture or at the offset of recognizable motion associated with the gesture. Onsets and offsets of gaze behavior were identified using visual cues from the individual's nose to assess direction of looking, their eyelids to onset and offset of gaze fixation, and their pupils when visible on the video. Coders used the step frame forward and backward controls in Noldus Observer XT to visualize changes in gaze behavior with greater clarity. Caregiver and infant communication acts were used in all subsequent analyses of dyadic synchrony and turn‐taking. 22.8% of videos (8 of 35) were independently coded for reliability of mother and infant communication and coordinated attention by a graduate student in speech‐language pathology. Reliability coding procedures mirror those described for infant attention. Caregiver and infant communication were each represented as binary time series, where presence of a communication behavior was coded as a 1 and absence of a communication behavior was coded as 0 across 600 0.5‐second windows. The resulting binary time series was used to derive all synchrony and turn‐taking variables, therefore reliability of communication events was calculated with a 0.5‐second tolerance window. Percent agreement for infant communication behavior was 94.7% and the Kappa agreement value for infant communication was *κ* = 0.69. Percent agreement for caregiver's communication behaviors was 88.7%, and *κ* = 0.77.

#### 2.2.3 Dyadic Interaction Measures

##### 2.2.3.1 Caregiver–Infant Synchrony

Interpersonal synchrony was measured using Cross‐Recurrence Quantification Analysis (CRQA) of caregiver and infant communicative behavior. CRQA is frequently used to estimate synchrony in nominal time series that are non‐linear in nature, such as behavioral coordination during naturalistic interaction (Markova and Nguyen 2022; Xu et al. 2020). Caregiver and infant communication behavior, which was coded as continuous timed‐event data, was converted to two nominal time series for both caregivers and infants by collapsing events into 0.5‐second partial‐event intervals using open source processing scripts provided by Xu et al. (2020) in MATLAB (The MathWorks Inc. 2022). The result was a binary time series of 600 potential events for caregivers and a separate time series of equal length for infants. Downsampling continuous timed‐event data to intervals as large as 1 second has been shown to effectively capture caregiver–child synchrony of communication behaviors (Beebe et al. 2010; Markova and Nguyen 2022; Van Keer et al. 2019). To avoid overinflating the recurrence of periods where neither caregiver nor infant are communicating, caregivers and infants received a distinct and separate “non‐event” code (Coco and Dale 2014). The lag was set to 3 seconds and the radius was set to 0.1. Synchrony was estimated through the recurrence rate of each dyad's interactive behavior, which was measured using the *crqa* function in R (Coco and Dale 2014). The recurrence rate, similar to a correlation between two time series, represents the percentage of recurrent events within a lag window (for further discussion, see Dale et al. 2011).

##### 2.2.3.2 Caregiver–Infant Turn‐Taking

Turn‐taking measures were calculated by identifying bouts of communication behaviors between the mother and infant with at least two turns (i.e., *infant–caregiver–infant* or *caregiver–infant–caregiver*). Two turns was selected as the lower boundary to avoid conflating turn‐taking with caregiver responsiveness or caregiver imitation, where caregivers are the primary contributor (Landry et al. 2006; McDuffie and Yoder 2010). Two‐turn bouts ensure that both the caregiver and infant are responsive to each other's communication acts within the bout and that our turn‐taking measure reflected a truly dyadic process. Each turn within a bout must occur within 3 seconds of each other, and all bouts must be separated by at least 3 seconds (Gratier et al. 2015; Van Egeren et al. 2001). While caregiver–infant synchrony measured the coordination between mother and infant communicative behaviors across the entire interaction, we also sought to specifically measure turn‐taking behaviors to assess how attention states were related to longer bouts of reciprocal communication that consisted of at least two turns. Proportion of codable time spent in bouts of turn‐taking was calculated to measure caregiver–infant turn‐taking, to take into account both frequency of turn‐taking bouts and the length of bouts.

### 2.3 Analysis Plan

A sensitivity power analysis revealed that the sample size of 35 dyads had sufficient power (0.8) to detect large effect sizes (*f* = 0.49) with *α* = 0.05. Assumptions of normality were tested using one‐sample Kolmogorov–Smirnov tests. Proportion of time spent in coordinated attention, caregiver–infant synchrony, and proportion of time in bouts of turn‐taking were all not normally distributed (*ps* < 0.001), therefore synchrony, turn‐taking, and attention variables were *z*‐transformed prior to all subsequent analyses. To assess the relationship between coordinated attention and caregiver–infant synchrony and turn‐taking, we conducted correlational analyses using Pearson correlations. Linear regression models were used to assess whether time spent in casual attention or focused attention was associated with synchrony and turn‐taking. All statistical analyses were computed using RStudio (RStudio Team 2022).

## 3 Results

Descriptive statistics of all attention and dyadic interaction variables are provided in Table 2. As expected, caregiver–infant synchrony and proportion of time spent in turn‐taking bouts were strongly correlated (*r*(33) = 0.75, *p* < 0.001). Infant chronological age was not associated with caregiver–infant synchrony (*p* = 0.98), turn‐taking (*p* = 0.26), or coordinated attention (*p* = 0.99). Similarly, infant language ability as measured on the REEL‐3 was not associated with caregiver–infant synchrony (*p* = 0.20), turn‐taking (*p* = 0.17), or coordinated attention (*p* = 0.062). Neither age nor language ability were included as covariates in subsequent analyses.

**TABLE 2:** Descriptive statistics for infant visual attention and dyadic interaction variables.

*N* = 35	Mean	SD	Range
Communication acts			
Caregiver vocalizations (%)	26.50	10.40	7.66–47.15
Caregiver gestures (%)	6.43	5.33	0–23.97
Infant vocalizations (%)	3.62	3.03	0–10.53
Infant gestures (%)	1.62	2.09	0–6.88
Infant eye gaze (%)	2.55	2.27	0–9.03
Visual attention			
Unfocused attention (%)	4.91	5.86	0–28.18
Focused attention (%)	38.35	26.69	0–89.24
Casual attention (%)	56.74	25.47	10.76–97.43
Dyadic interaction			
Coordinated attention (%)	84.99	10.89	44.14–98.85
Caregiver–infant synchrony (RR)	3.46	2.97	0.095–10.42
Time spent in bouts of turn‐taking (%)	27.93	17.82	1.39–72.46

Abbreviations: %, percentage of codable time; RR, recurrence rate of mother and infant communication.

### 3.1 RQ1: Relationship Between Coordinated Attention and Measures of Dyadic Interaction

To examine our first research question, whether caregiver–infant coordinated attention was associated with dyadic synchrony or turn‐taking, we calculated Pearson correlations. Caregiver–infant synchrony was operationalized as the recurrence rate from CRQA analyses of caregiver–infant communication, and turn‐taking was measured as the proportion of time spent in bouts of turn‐taking. Contrary to our hypothesis, proportion of time spent in coordinated attention was not associated with measures of caregiver–infant dyadic interaction. Proportion of time in coordinated attention was not associated with either caregiver–infant synchrony (*r*(33) = 0.13, *p* = 0.46) nor proportion of time spent in bouts of turn‐taking (*r*(33) = 0.15, *p* = 0.38). Of note, while infants were variable in their attentional states across the sample, rates of coordinated attention during play were high overall, with a mean proportion of time spent in coordinated attention of approximately 85%.

### 3.2 RQ2: Do Infant Casual or Focused Attention Predict Dyadic Interaction Measures?

To examine our second research question, whether infant casual attention or focused attention was associated with higher rates of caregiver–infant synchrony and turn‐taking, we conducted general linear models. Prior to running the linear models, a Grubbs test for outliers was completed to identify and remove any statistical outliers. One outlier was detected (*p* < 0.001) and that participant was removed from further analyses. All variables were *z*‐transformed and models were assessed for linearity and homoscedasticity through visual inspection by plotting residuals against the fitted model.

**FIGURE 2:**
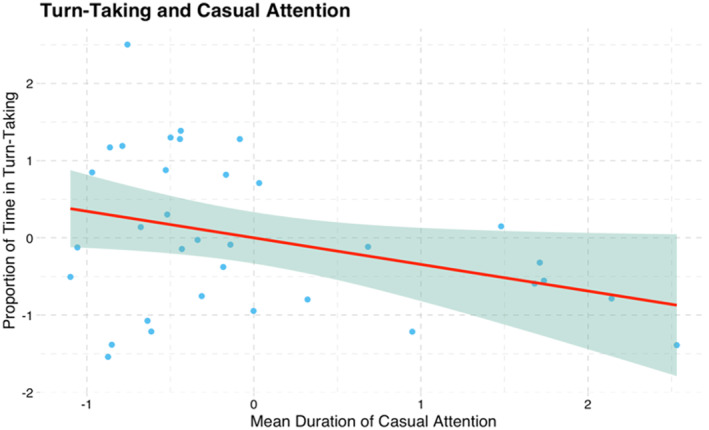
Relationship between mean duration of infant casual attention (*z*‐transformed) and proportion of time spent in turn‐taking bouts (*z*‐transformed).

Neither caregiver–infant synchrony (*t*(33) =  = −0.20, *p* = 0.25) nor time spent in bouts of turn‐taking (*t*(33) =  = −0.12, *p* = 0.497) were significantly associated with mean duration of focused attention. Associations between caregiver–infant synchrony and mean duration of casual attention were nonsignificant (*t*(33) = −1.61, *β* = 0.76, *p* = 0.12). Proportion of time spent in bouts of turn‐taking, on the other hand, was significantly associated with mean duration of casual attention (*t*(33) = −2.08, *β* = 0.71 *p* = 0.046) (see Figure 2). Infants who demonstrated shorter spurts of casual attention, resulting in a shorter mean duration of casual attention, spent significantly more time in bouts of turn‐taking with their caregiver.

### 3.3 Exploratory Analysis: Influence of Caregiver Communication on Attention and Dyadic Interaction

**FIGURE 3:**
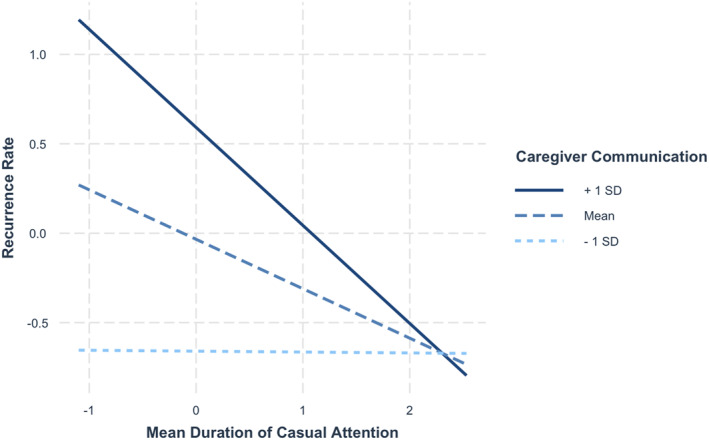
Interaction of time caregiver's spent communicating and mean duration of infant casual attention (*z*‐transformed) on caregiver–infant synchrony recurrence rate.

Based on the our results and the findings of Schroer and Yu (2022), which reported that infants whose parents were less talkative during parent–child play demonstrated longer durations of sustained attention and joint attention, we conducted exploratory interaction models examining how caregiver communication moderated the relationship between infant casual attention and caregiver–child synchrony and turn‐taking. We first examined whether the effect of infant casual attention on caregiver–child synchrony differed across dyads varying in amount of caregiver communication. A moderated multiple regression analysis demonstrated a significant main effect of amount of caregiver communication (*b* = 4.96, SE = 1.01, *t* = 4.90, *p* < 0.001) and a significant interaction effect (*b* = −2.16, SE = 0.99, *t* = −2.16, *p* = 0.039), indicating that the effect of infant casual attention on caregiver–child synchrony was stronger in caregivers who communicated more (see Figure 3). We then examined whether the effect of infant casual attention on turn‐taking differed across dyads varying in amount of caregiver communication. A moderated multiple regression analysis revealed a significant main effect of amount of caregiver communication on proportion of time in turn‐taking bouts (*b* = 2.87, SE = 1.24, *t* = 2.32, *p* = 0.027), though the interaction effect was not significant (*b* = −1.65, SE = 1.22, *t* = −1.36, *p* = 0.18).

## 4 Discussion

### 4.1 Role of Coordinated Attention in Dyadic Interaction

The first aim of the current study was to determine the relationship between coordinated attention and measures of mother–infant synchrony and turn‐taking. Based on previous work describing the relationship between coordinated attention and language skills such as vocabulary (Abney et al. 2020; Yu and Smith 2016), we hypothesized that synchrony and turn‐taking would be positively associated with coordinated attention. Periods of synchrony and turn‐taking during play may be important contexts for social communication and language learning that can be facilitated through coordinated attention with a caregiver; therefore we expected that both synchrony and turn‐taking would be associated with coordinated attention. Further, coordinated attention requires that both caregiver and infant are attending to the same object or activity, which could serve as a shared referent for communication, therefore, the more opportunities infants and mothers had to focus on the same activity, the more opportunities they might have to communicate about it. Surprisingly, proportion of time spent in coordinated attention was not related to either synchrony or time spent in turn‐taking bouts. One possible explanation for these findings is that time spent in coordinated attention tended to be relatively high among dyads, while amount of turn‐taking and communicative behavior overall was more varied among dyads. Mothers and infants were sitting face to face, and we also informally observed that mothers tended to pull out one toy at a time, whereas other play configurations such as floor play may naturally lead to more distractibility and less coordinated attention. While all dyads spent at least 40% of their interaction in coordinated attention, 8 of 35 dyads spent less than 10% of time in bouts of turn‐taking, which may have dampened any associations between a relatively high incidence attention state and relatively low incidence turn‐taking behaviors during the interaction. Additionally, other variables such as parent responsiveness, affect, or social communication skills may have driven differences in dyadic interaction variables above and beyond coordinated attention. Future studies with well‐powered samples could investigate the moderating effect of these variables on the relationship between coordinated attention and interaction dynamics.

### 4.2 Casual Attention Predicts Time Spent in Turn‐Taking During Play

The second aim of the current study was to assess the relationship between infant visual attention states and dyadic interaction patterns. Mean duration of focused attention was not associated with either caregiver–infant synchrony or time spent in bouts of turn‐taking during play. While previous research has linked linking longer sustained attention to objects to better language skills later in development (Salley et al. 2013; Yu et al. 2019), longer duration of focused attention was not related to concurrent dyadic interaction dynamics during naturalistic play. Mean duration of casual attention, on the other hand, was significantly negatively associated with time spent in bouts of turn‐taking. These findings suggest that shorter periods of infant casual attention were associated with more time spent in turn‐taking with their caregiver. Notably, Salley et al. (2013) and Yu et al. (2019) both assessed sustained (i.e., focused) attention in slightly younger infants (11 and 9 months, respectively). It is possible that language skills are more tightly linked to sustained attention in the first year of life and then casual attention as infants enter the second year of life, though further longitudinal research across both proximal and distal language outcomes is needed to directly test this hypothesis.

Both synchrony and turn‐taking exhibited a negative relationship with duration of casual attention, though the relationship between casual attention and caregiver–child synchrony did not reach significance. These findings are consistent with Gartstein et al. (2008), who found that longer durations of infant visual attention were associated with lower ratings of synchrony/reciprocity during interaction. However, Gartstein et al. (2008) measured overall duration of orienting to visual stimuli, while the current study sought to account for flexibility and shifting attention by differentiating between casual and focused attention. It is possible that even long periods of casual attention may not promote increased communicative interaction with caregivers, and that ability to shift attention should be discretely measured in future work.

One possible interpretation of these findings is that mothers whose infants demonstrated shorter periods of attention during play were more likely to capitalize on those moments of attention by initiating communication with their infant or responding to their infants' communicative behaviors than mothers whose infants demonstrated longer periods of attention, thus facilitating more turn‐taking bouts. This interpretation is consistent with findings that the moderating effect of attention on the relationship between parental responsiveness and infant vocabulary skills was stronger in parents who were less talkative (Schroer and Yu 2022), suggesting that less talkative parents may be more selective in identifying opportunities to extend their infants attention to support word learning. To test this hypothesis, we conducted exploratory moderated multiple regression analyses assessing the impact of caregiver communication behavior on the relationship between casual attention and dyadic interaction. We found that the relationship between caregiver–child synchrony, but not turn‐taking, and shorter bouts of casual attention was stronger in dyads where caregivers spent more time communicating. These findings, in contrast to Schroer and Yu (2022), indicate that more communicative parents may have driven variability in caregiver–child synchrony, particularly with infants who showed shorter bouts of casual attention. Dyads with less communicative caregivers, on the other hand, demonstrated no relationship between casual attention and synchrony, highlighting the important role of caregivers in shaping and scaffolding social interaction at this age. Due to the small sample size and variability in infant age in the current study, more research on the moderating effects of caregiver behavior on infant attention in dyadic interaction is needed before drawing conclusions about this complex relationship. An alternative explanation is that the positive vocabulary outcomes associated with sustained attention in previous studies (e.g., Yu et al. 2019) may not be facilitated through reciprocal interaction, and that longer periods of turn‐taking or communicative interaction are not necessary for infant vocabulary learning during play, but rather reflect other developmentally important social learning processes.

### 4.3 Limitations and Future Directions

This study expands on previous work exploring the role of attention in language development in two important ways: (1) by differentiating between focused and casual attentional states, and (2) by using behavioral measures of dyadic interaction dynamics rather than third‐party ratings. However, there are multiple limitations to consider when interpreting the current findings. One limitation of the current study is that we may have been underpowered to detect significant effects, particularly between caregiver–infant synchrony and duration of casual attention. Because synchrony and time spent in bouts of turn‐taking were so strongly correlated (*r* = 0.75), it was surprising that turn‐taking was significantly associated with duration of casual attention while synchrony was not. Given the significant interaction effect of caregiver communication on infant casual attention and synchrony, it is possible that variability in caregiver communication behavior during the play sample dampened the effects of infant attention on dyadic interaction. It is also possible that associations between interactive variables and other attentional states, such as focused attention, may have emerged in a larger sample of dyads or during longer play samples. On a related note, interrater reliability for infant communicative behaviors (*Κ* = 0.69) was lower than standard thresholds for reliability (e.g., *K* = 0.7 or higher). While our interrater reliability was consistent with prior work using similar coding procedures (Lavelli and Fogel 2005; Van Keer et al. 2019), this degree of measurement error may have reduced our power to detect effects.

Additionally, the current sample represents a wide age range of infants in their second year of life, from approximately 11–23 months. While chronological age was not associated with the dyadic outcome measures, results should be interpreted with the knowledge that infants across the sample presented a range of developmental skills across language and motor milestones.

While mean duration of casual attention was significantly associated with time spent in bouts turn‐taking, it is possible that other discrete measures of infant attentional skills (e.g., distractibility, social vs. object attention, or inhibitory control) or caregiver behaviors such as caregiver‐initiated joint attention may better characterize the contributions of attention to social interaction. For example, while the face‐to‐face unstructured play interaction utilized in the current study was social in nature, we did not measure the amount of object focused play compared to socially focused play, which may influence the relationship between casual attention and measures of dyadic interaction. Future studies should also examine infant visual attention and interaction dynamics during multiple social contexts and play configurations. The play sample in the current study was completed in a high chair with caregivers sitting face to face with their child, which affords different opportunities and constraints for visual attention than unstructured floor play, in‐home routines, or shared book reading, for example (Kretch 2025; Schneider et al. 2022). Results of the current study should be interpreted with the knowledge that the play sample occurred in a reduced‐distraction environment and that children had limited opportunity for movement.

Finally, we used both mother–infant synchrony and time spent in bouts of turn‐taking as proxies for reciprocal interaction, though other aspects of reciprocal social interaction could be measured and analyzed in relation to visual attention in future studies. We believe that synchrony and turn‐taking were well‐suited to capture relationships with periods of attention because they tap into the temporal relationships between mother and infant communication. Future work could expand on these measures by examining different forms of mothers' speech (e.g., comments vs. directives) or the semantic contingency between mother and infant communication during turn‐taking.

### 4.4 Conclusions

Overall, our findings suggest that infants' shorter bouts of casual attention, but not focused attention, were associated with turn‐taking with their caregiver. This finding provides new insight into how patterns of visual attention relate to social interaction dynamics, with implications for social communication and language learning. We also found preliminary evidence for the moderating relationship of caregiver communication on shorter bouts of casual attention and caregiver–infant synchrony which can be further disentangled in future work. Further, coordinated attention alone was not associated with levels of caregiver–infant synchrony or turn‐taking, suggesting that other factors such as responsiveness or semantic contingency in the context of coordinated attention may be more closely related to dyadic interaction variables. Future work could continue to measure such factors along with individual visual attention skills such as alternating gaze or switching attention to tease apart the underlying processes by which attention supports social communication during interaction.

## Author Contributions

**Olivia Boorom:** conceptualization, methodology, formal analysis, writing – original draft. **Brenda Salley:** methodology, investigation, writing – review and editing, supervision, project administration, funding acquisition.

## Ethics Statement

The study was conducted in accordance with the ethical standards of the American Psychological Association and approved by the Human Subjects Committee at the University of Kansas Medical Center (#2932).

## Consent

All caregivers provided informed consent at the time of enrollment.

## Conflicts of Interest

The authors declare no conflicts of interest.

## Data Availability

The data that support the findings of this study are available on request from the corresponding author. The data are not publicly available due to privacy or ethical restrictions.
